# Municipal Governments' Long‐Term Care Prevention Efforts and Homebound Status of Older Adults: A Three‐Year Cohort Study in Japan

**DOI:** 10.1111/ggi.70209

**Published:** 2025-10-15

**Authors:** Duc Sy Minh Ho, Kaori Yamaguchi, Kazushige Ide, Sakura Kiuchi, Naoki Kondo, Jun Aida

**Affiliations:** ^1^ Department of Dental Public Health, Graduate School of Medical and Dental Sciences Institute of Science Tokyo Tokyo Japan; ^2^ Faculty of Odonto‐Stomatology, Hue University of Medicine and Pharmacy Hue University Hue Vietnam; ^3^ Department of Health and Welfare Services National Institute of Public Health Saitama Japan; ^4^ Department of Community Building for Well‐Being, Center for Preventive Medical Sciences Chiba University Chiba Japan; ^5^ Department of International and Community Oral Health, Graduate School of Dentistry Tohoku University Miyagi Japan; ^6^ Frontier Research Institute for Interdisciplinary Sciences Tohoku University Miyagi Japan; ^7^ Department of Social Epidemiology, Graduate School of Medicine Kyoto University Kyoto Japan

**Keywords:** daily living support system development progress, homebound status, long‐term care prevention and daily living support progress, municipal governments' long‐term care prevention efforts

## Abstract

**Aim:**

Homebound status poses considerable risks for adverse health outcomes among older adults. To enhance the comprehensive care system, municipalities have implemented efforts aimed at long‐term care prevention. It is assumed that such efforts may help mitigate homeboundness. Therefore, this study examined the association between municipal long‐term care prevention efforts and homebound status among older adults.

**Methods:**

This 3‐year cohort study utilized panel data between 2019 and 2022 from the Japan Gerontological Evaluation Study (JAGES), involving adults aged ≥ 65 years who were not homebound in 2019. As proxies for municipalities' prevention efforts, the Daily Living Support System Development Progress (DLSSDP) scores and Long‐term Care Prevention and Daily Living Support Progress (LCPDLSP) scores in 2019 were used as explanatory variables. The outcome was homebound status in 2022. Individual‐ and municipality‐level characteristics in 2019 were included as confounders. Multilevel logistic regression with multiple imputation was performed to estimate odds ratios for the association between each score and homebound status.

**Results:**

The study included 89 914 participants (female: 51.6%, mean age: 76.5 years). The overall incidence of homebound status in 2022 was 2.8%, with variations ranging from 1.3% to 6.4% across 49 municipalities. Compared with older adults in municipalities with high DLSSDP scores, those in municipalities with low scores had significantly higher odds of being homebound (odds ratio: 1.22; 95% confidence interval: 1.04–1.44). There were no significant differences in homebound odds across municipalities with varying LCPDLSP scores.

**Conclusion:**

Living in municipalities with higher DLSSDP scores was associated with not being homebound.

## 1 Introduction

Homebound status is a risk factor for various health problems and functional disabilities among older adults [1, 2]. It often leads to social isolation and reduced physical activity [3], which are associated with limited social interaction. Over time, being homebound can result in serious health consequences, including diminished quality of life [4] and increased mortality [5, 6].

Homebound status can be influenced by both individual‐ and community‐level factors [7, 8]. Social participation may help alleviate it by encouraging engagement and reducing isolation. In rural Japan, older adults in communities with abundant civic engagement had lower homebound prevalence [8]. Consequently, municipal policies that promote social participation can help create supportive community environments and mitigate homeboundness. Japan has introduced initiatives [9] such as community gathering places to enhance social participation, which have been demonstrated to improve older adults' health [10, 11, 12, 13]. Such community‐level interventions may contribute to regional differences in homebound prevalence.

Thus, municipal long‐term care prevention efforts may help create favorable environments and reduce homebound status. This study examined two proxies for such efforts: the Long‐term Care Prevention and Daily Living Support Progress (LCPDLSP) and the Daily Living Support System Development Progress (DLSSDP). These initiatives aim to address older adults' daily living needs, such as enhancing older people's clubs and providing shopping assistance [14, 15]. Although the LCPDLSP covers a broad range of measures, including service evaluation and resident engagement, the DLSSDP focuses specifically on the roles of living support coordinators and consultative bodies in prevention activities [14, 15, 16]. Participation in these efforts is mandatory nationwide, with municipalities required to report their implementation progress to the Japanese government, which allocates financial incentives accordingly [17]. Although international studies have explored government support for homebound older adults—such as housing and transportation in the United States [18] and healthcare and social services in Sweden [19]—most have focused on providing care to those already homebound rather than on preventive approaches. To address this gap, the present study investigated the association between the LCPDLSP and DLSSDP scores, as proxies for certain long‐term care prevention efforts carried out by municipal governments, and homebound status among older adults in Japan.

## 2 Materials and Methods

### 2.1 Data Collection

This three‐year cohort study used panel data from the Japan Gerontological Evaluation Study (JAGES), conducted between 2019 and 2022, targeting individuals aged 65 years and older. The JAGES is a population‐based study conducted across multiple municipalities that examines social determinants of health and preventive care strategies for older adults who are not eligible for public long‐term care insurance benefits [9, 20].

This dataset has a two‐level hierarchical structure comprising individuals and municipalities. In Japan, municipalities are local government units directly below prefectures [21]. The JAGES surveys in 2019 and 2022 were distributed to eligible residents in 49 municipalities across the country.

**FIGURE 1:**
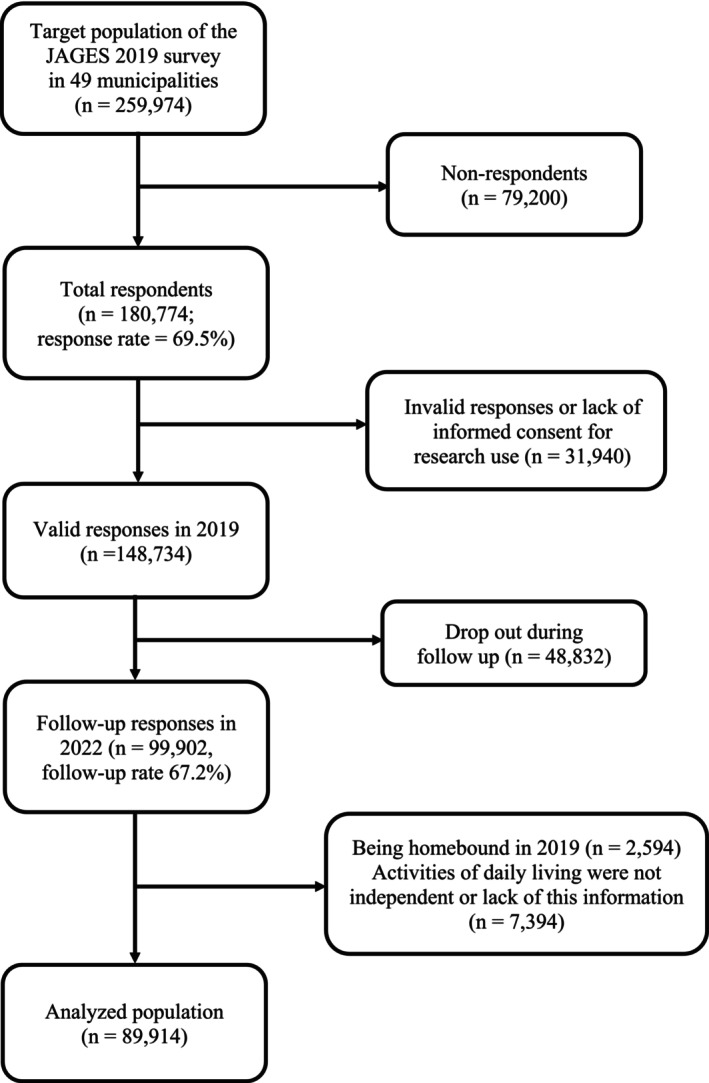
Flowchart of the study participants.

In 2019, the JAGES conducted a comprehensive self‐reported questionnaire survey targeting 259 974 older adults. A total of 180 774 individuals responded, yielding a response rate of 69.5%. Of these, 31 940 were excluded due to invalid responses or lack of informed consent for research purposes, leaving 148 734 valid responses. In 2022, 99 902 individuals who had previously participated in the survey responded, resulting in a follow‐up rate of 67.2%. We further excluded individuals who required assistance with basic daily activities, such as bathing or using the toilet, as well as those with missing information regarding such assistance (*n* = 7394). Additionally, 2594 older adults who were already homebound in 2019 were excluded. Consequently, our analysis included a total of 89 914 older adults from 49 municipalities. A flowchart of the participants is shown in Figure 1.

### 2.2 Outcome Variable

In this study, the outcome variable—homebound status in 2022—was determined based on responses to the question: “How often do you go out (including visits to the field, neighborhood, hospital, or shopping)?” Participants selected from seven options: “Five times a week or more,” “Four times a week,” “Two to three times a week,” “Once a week,” “One to three times a month,” “Several times a year,” and “None.” Respondents were classified as homebound if they selected any of the last three options. Homebound status was dichotomized into going out of the house once a week or more versus less than once a week. This definition has been widely adopted in previous studies [8, 22, 23, 24]. Its predictive validity for functional disability has also been confirmed, supporting its reliability [2, 25].

### 2.3 Explanatory Variables

The explanatory variables were the LCPDLSP and DLSSDP scores for fiscal year 2019, which served as proxies for specific municipal long‐term care prevention efforts aimed at strengthening the comprehensive care system for older adults [14, 16]. These two municipality‐level variables were considered relevant to homebound prevention and were obtained from the Summary of Grants for the Strengthening Insurers' Functions for fiscal year 2019 [26]. The LCPDLSP scores reflect a wide range of activities that promote long‐term care prevention and community support. These scores comprised eight indicators, including information dissemination, verification of service quantities, stakeholder consultation, service provision based on older adults' needs, participation rate in community gathering places, provision of community resource information, involvement of rehabilitation professionals, and promotion of resident engagement in prevention activities [16]. The DLSSDP scores primarily reflect the roles of the living support coordinators and consultative bodies. These scores comprised four indicators: municipal support for living support coordinators, initiatives by coordinators, initiatives by consultative bodies, and resource development for older adults through the activities of coordinators and consultative bodies [16]. The detailed evaluation criteria and distributions of the raw LCPDLSP and DLSSDP scores are shown in Tables S1 and S2; Figures S1 and S2, respectively. As the raw score distributions were non‐normal, we categorized both scores into three levels based on participant tertiles to achieve approximately equal group sizes. The LCPDLSP scores (0–89) were classified into three levels: low (0–71), medium (72–75), and high (76–89). Similarly, the DLSSDP scores (0–46) were classified into three levels: low (0–35), medium (36–45), and high (46).

### 2.4 Confounding Variables

The analysis was adjusted for both individual‐ and municipality‐level confounders. Individual characteristics, based on a previous study [8], consisted of age, sex, living arrangements, marital status, educational attainment, equivalent annual household income, depressive symptoms, self‐rated health, and number of medical diseases under care or sequelae. Municipality‐level confounders included population density [27] and average income [28]. Detailed classifications of these confounders are provided in the Supplementary Methods.

### 2.5 Statistical Analysis

Descriptive analyses were performed to examine the incidence of homebound status in 2022 by baseline (2019) demographic characteristics of the participants. Subsequently, a multilevel logistic regression analysis with a robust variance estimator was applied to estimate the odds ratios of LCPDLSP and DLSSDP score levels for homebound status in 2022 using the Stata command “melogit”. The analysis comprised four models based on a two‐level hierarchical structure with individuals nested within municipalities. Model 1 was a univariable model in which each individual‐ and municipality‐level characteristic was analyzed separately. Model 2 simultaneously adjusted for all individual‐ and municipality‐level confounders. Model 3 examined the association between LCPDLSP score levels (explanatory variable) and homebound status in 2022 (outcome), adjusting for all confounders. Model 4 used the same outcome and confounders as Model 3 but replaced the explanatory variable with DLSSDP score levels. The variance partition coefficient (VPC) was also calculated for each model, except for the univariable model. To confirm the consistency of the results, a supplementary stratified analysis was conducted based on municipal population density.

The numbers and proportions of missing variables are shown in Table S3. Multiple imputation was used to reduce bias, assuming a “missing at random” mechanism to address missing data [29]. Twenty imputed datasets were produced using multiple imputation by chained equations. The variables used in the imputation comprised explanatory and outcome variables, as well as the confounders [30]. Rubin's rule was applied to derive the pooled estimates [31].

As part of our sensitivity analyses, we first conducted a complete case analysis, including only participants with no missing data. Supplementary sensitivity analyses were undertaken using the LCPDLSP and DLSSDP scores for fiscal years 2020 [32] and 2021 [33] as explanatory variables to examine their associations with homebound status in 2022. These analyses employed the same dataset and analytical framework as the primary analysis. The raw score distributions are presented in Figures S3–S6, respectively. Stata (version 18.0; StataCorp LLC, College Station, TX, USA) was used for all analyses. Statistical significance was set at two‐tailed *p* < 0.05.

Ethical Approval for the secondary data analysis was granted by the Ethics Review Committee of Tokyo Medical and Dental University, Japan (approval no. D2022‐040), and the Ethics Committee on Research of Human Subjects at Chiba University Graduate School of Medicine (approval no. M10460). Only data from participants who provided informed consent were used.

## 3 Results

**FIGURE 2:**
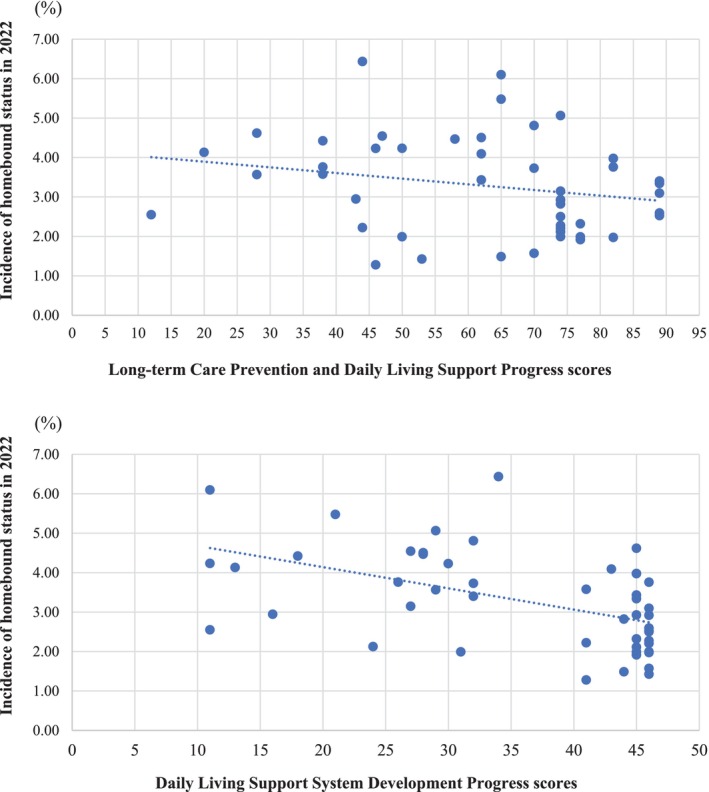
Incidence of homebound status between 2019 and 2022 based on the 2019 daily living support system development progress and long‐term care prevention and daily living support progress scores, stratified by municipalities after multiple imputation (*n* = 89 914).

A total of 89 914 older adults were included in the analysis. The participants' mean age was 76.5 years (SD = 5.7), with 51.6% being female. The overall homebound incidence in 2022 was 2.8%, varying between 1.3% and 6.4% across 49 municipalities. The incidence of homebound status between 2019 and 2022 based on the LCPDLSP and DLSSDP scores, stratified by municipalities after multiple imputation, is shown in Figure 2. Participants living in municipalities with high DLSSDP scores tended to have a lower incidence of homebound status. In comparison, participants in municipalities with higher LCPDLSP scores showed only a slight tendency toward a lower incidence of homebound status, and the association was weaker.

The incidence of homebound status in 2022 by baseline (2019) demographic characteristics among participants who were not homebound at baseline, after multiple imputation, is presented in Table 1. The corresponding table presenting column percentages is shown in Table S4. The incidence of being homebound increased with older age, living alone, lower educational attainment, lower household income, more severe depressive symptoms, poorer self‐rated health, and more medical conditions. It was also higher in municipalities with lower population density, lower average income, and worse DLSSDP scores. The incidences of being homebound in the municipalities with high, middle, and low DLSSDP score levels were 2.3%, 2.7%, and 3.6%, respectively. For LCPDLSP score levels, the corresponding incidences were 2.7%, 2.5%, and 3.1%, respectively. Tables S5 and S6, which present the demographic characteristics of participants who were not homebound at baseline (2019) after multiple imputation, stratified by LCPDLSP and DLSSDP score levels, respectively, also show similar results.

**TABLE 1:** Incidence of homebound status in 2022 by baseline (2019) demographic characteristics among participants who were not homebound at baseline, after multiple imputation (*n* = 89 914).

Characteristics	Incidence of homebound status in 2022		
	Going out of the house once a week or more	Going out of the house less than once a week	Total
	(row%)^a^	(row%)^a^	No. (col%)^b^
	(*n* = 87 407)	(*n* = 2507)	(*n* = 89 914)
Individual‐level variables			
Age			
65–69	98.5%	1.5%	24 150 (26.9%)
70–74	98.2%	1.8%	28 419 (31.6%)
75–79	97.1%	2.9%	22 269 (24.8%)
80–84	94.9%	5.1%	11 196 (12.5%)
≥ 85	89.3%	10.7%	3880 (4.2%)
Sex			
Male	97.2%	2.8%	43 520 (48.4%)
Female	97.3%	2.7%	46 394 (51.6%)
Living arrangements			
Living with others	97.3%	2.7%	77 229 (85.9%)
Living alone	96.9%	3.1%	12 685 (14.1%)
Marital status			
With a spouse	97.5%	2.5%	68 218 (75.9%)
Without a spouse	96.3%	3.7%	21 696 (24.1%)
Educational attainment			
≥ 10 years	97.8%	2.2%	68 556 (76.2%)
< 10years	95.5%	4.5%	21 358 (23.8%)
Equivalent annual household income			
≥ 4.0 million yen	98.3%	1.7%	11 431 (12.7%)
≥ 1.0 and < 4.0 million yen	97.4%	2.6%	68 757 (76.5%)
< 1.0 million yen	94.4%	5.7%	9727 (10.8%)
Depressive symptoms^c^			
No depression	97.7%	2.3%	72 310 (80.4%)
Mild depression	95.6%	4.4%	14 304 (15.9%)
Severe depression	94.4%	5.6%	3300 (3.7%)
Self‐rated health			
Good	97.5%	2.5%	81 693 (90.9%)
Poor	94.3%	5.7%	8221 (9.1%)
Number of medical diseases under care or sequelae			
0	98.1%	1.9%	17 954 (20.0%)
1	97.3%	2.7%	32 532 (36.2%)
2	97.0%	3.0%	23 203 (25.8%)
≥ 3	96.3%	3.7%	16 225 (18.0%)
Municipality‐level variables			
Population density			
Metropolitan	97.7%	2.3%	29 651 (33.0%)
Urban	97.7%	2.3%	26 184 (29.1%)
Rural	96.4%	3.6%	34 079 (37.9%)
Municipality‐level average income			
≥ 3800 thousand yen/person	97.7%	2.3%	30 369 (33.8%)
≥ 2900 and < 3800 thousand yen/person	97.6%	2.4%	29 506 (32.8%)
< 2900thousand yen/person	96.3%	3.7%	30 039 (33.4%)
LCPDLSP^d^ score levels			
High	97.3%	2.7%	19 811 (22.0%)
Middle	97.5%	2.5%	38 599 (43.0%)
Low	96.9%	3.1%	31 504 (35.0%)
DLSSDP^e^ score levels			
High	97.7%	2.3%	37 052 (41.2%)
Middle	97.3%	2.7%	26 062 (29.0%)
Low	96.4%	3.6%	26 800 (29.8%)

^a^ Row percentage.

^b^ Column percentage.

^c^ We used the 15‐item Geriatric Depression Scale (GDS‐15) to classify the depressive symptoms.

^d^ 2019 Long‐term Care Prevention and Daily Living Support Progress scores (0–89) of the municipalities were classified into three levels: low (0–71), medium (72–75), and high (76–89), based on the tertiles of participants.

^e^ 2019 daily living support system development progress scores (0–46) of the municipalities were classified into three levels: low (0–35), medium (36–45), and high (46), based on the tertiles of participants.

The association between individual‐ and municipality‐level factors and homebound status among older adults across municipalities, analyzed using multilevel logistic regression after multiple imputation, is presented in Table 2. After adjusting for all confounders, the multilevel logistic regression analysis revealed that, compared with older adults in municipalities with high DLSSDP scores, those in municipalities with low scores had 1.22 times higher odds (95% CI: 1.04–1.44) of being homebound. No significant differences in the odds of being homebound were observed across municipalities with varying LCPDLSP scores. The VPC of the null model, which did not include dependent variables, was 3.35%. After adjusting for confounders, the variation in homebound status across municipalities decreased, and the VPC dropped to 0.98% (Model 2). The VPCs of Models 3 and 4 were 0.84% and 0.86%, respectively. These results suggest that over 10% of the inter‐municipality variation in homebound status was explained by LCPDLSP or DLSSDP score levels.

**TABLE 2:** Association between individual‐ and municipality‐level factors and homebound status among older adults across municipalities using multilevel logistic regression after multiple imputation (*n* = 89 914).

	Model 1^a^		Model 2^b^		Model 3^c^		Model 4^d^	
	OR^e^	95% CI^f^	OR^e^	95% CI^f^	OR^e^	95% CI^f^	OR^e^	95% CI^f^
Fixed effects								
Explanatory variables								
LCPDLSP^g^ score levels								
High	1.00	Reference			1.00	Reference		
Middle	0.94	0.76; 1.18			0.86	0.73; 1.02		
Low	1.20	0.95; 1.52			1.00	0.83; 1.20		
DLSSDP^h^ score levels								
High	1.00	Reference					1.00	Reference
Middle	1.17	0.94; 1.46					1.15	0.97; 1.37
Low	1.66	1.36; 2.03					1.22	1.04; 1.44
Individual‐level confounders								
Age								
65–69	1.00	Reference	1.00	Reference	1.00	Reference	1.00	Reference
70–74	1.26	1.10; 1.45	1.18	1.02; 1.36	1.18	1.02; 1.37	1.18	1.03; 1.37
75–79	2.12	1.83; 2.47	1.84	1.57; 2.15	1.84	1.57; 2.15	1.84	1.58; 2.15
80–84	3.71	3.18; 4.33	3.05	2.57; 3.62	3.05	2.57; 3.62	3.06	2.58; 3.63
≥ 85	8.09	7.03; 9.32	6.38	5.59; 7.29	6.39	5.60; 7.29	6.40	5.60; 7.30
Sex								
Male	1.00	Reference	1.00	Reference	1.00	Reference	1.00	Reference
Female	0.96	0.84; 1.09	0.92	0.82; 1.03	0.91	0.81; 1.03	0.92	0.82; 1.03
Living arrangements								
Living with others	1.00	Reference	1.00	Reference	1.00	Reference	1.00	Reference
Living alone	1.13	0.95; 1.34	0.79	0.65; 0.95	0.79	0.65; 0.95	0.79	0.65; 0.96
Marital status								
With a spouse	1.00	Reference	1.00	Reference	1.00	Reference	1.00	Reference
Without a spouse	1.47	1.21; 1.68	1.22	1.07; 1.39	1.21	1.07; 1.38	1.21	1.07; 1.38
Educational attainment								
≥ 10 years	1.00	Reference	1.00	Reference	1.00	Reference	1.00	Reference
< 10 years	2.03	1.77; 2.33	1.31	1.15; 1.49	1.31	1.15; 1.49	1.31	1.15; 1.48
Equivalent annual household income								
≥ 4.0 million yen	1.00	Reference	1.00	Reference	1.00	Reference	1.00	Reference
≥ 1.0 and < 4.0 million yen	1.51	1.31; 1.75	1.30	1.13; 1.50	1.30	1.13; 1.50	1.30	1.13; 1.50
< 1.0 million yen	3.21	2.69; 3.82	2.01	1.70; 2.38	2.02	1.70; 2.39	2.01	1.70; 2.38
Depressive symptoms^i^								
Non	1.00	Reference	1.00	Reference	1.00	Reference	1.00	Reference
Mild	1.90	1.71; 2.12	1.61	1.43; 1.82	1.61	1.43; 1.82	1.61	1.42; 1.82
Severe	2.40	2.06; 2.79	1.81	1.50; 2.18	1.81	1.50; 2.18	1.81	1.50; 2.18
Self‐rated health								
Fair	1.00	Reference	1.00	Reference	1.00	Reference	1.00	Reference
Poor	2.32	2.07; 2.60	1.66	1.46; 1.88	1.66	1.46; 1.88	1.66	1.46; 1.88
Number of medical diseases under care or sequelae								
0	1.00	Reference	1.00	Reference	1.00	Reference	1.00	Reference
1	1.42	1.20; 1.67	1.18	1.00; 1.39	1.17	1.00; 1.39	1.18	1.00; 1.39
2	1.58	1.32; 1.88	1.18	0.98; 1.42	1.18	0.98; 1.42	1.18	0.98; 1.42
≥ 3	1.98	1.63; 2.40	1.26	1.02; 1.54	1.26	1.02; 1.54	1.26	1.02; 1.55
Municipality‐level confounders								
Population density								
Metropolitan	1.00	Reference	1.00	Reference	1.00	Reference	1.00	Reference
Urban	0.99	0.82; 1.19	0.95	0.68; 1.32	0.86	0.61; 1.20	0.93	0.67; 1.28
Rural	1.69	1.49; 1.91	1.28	0.88; 1.86	1.19	0.82; 1.72	1.20	0.82; 1.76
Municipality‐level average income								
≥ 3800 thousand yen/person	1.00	Reference	1.00	Reference	1.00	Reference	1.00	Reference
≥ 2900 and < 3800 thousand yen/person	1.08	0.89; 1.30	1.00	0.70; 1.41	1.04	0.74; 1.48	0.95	0.69; 1.30
< 2900 thousand yen/person	1.76	1.51; 2.05	1.16	0.76; 1.77	1.18	0.78; 1.78	1.08	0.73; 1.62
VPC^j^ (%)			0.98		0.84		0.86	

^a^ Univariable model.

^b^ Adjusted for age, sex, living arrangements, marital status, educational attainment, equivalent annual household income, depressive symptoms, self‐rated health, number of medical diseases under care or sequelae, population density, municipality‐level average income.

^c^ Adjusted for age, sex, living arrangements, marital status, educational attainment, equivalent annual household income, depressive symptoms, self‐rated health, number of medical diseases under care or sequelae, population density, and municipality‐level average income. 2019 Long‐term Care Prevention and Daily Living Support Progress score levels were included as explanatory variables.

^d^ Adjusted for age, sex, living arrangements, marital status, educational attainment, equivalent annual household income, depressive symptoms, self‐rated health, number of medical diseases under care or sequelae, population density, and municipality‐level average income. 2019 Daily Living Support System Development Progress score levels were included as explanatory variables.

^e^ Odds ratio.

^f^ Confidence interval.

^g^ Long‐term care prevention and daily living support progress.

^h^ Daily living support system development progress.

^i^ We used the 15‐item geriatric depression scale (GDS‐15) to classify the depressive symptoms.

^j^ Variance partition coefficient.

The results of the supplementary stratified and sensitivity analyses were consistent with those of the main analysis. Detailed findings are presented in the Supplementary Results.

## 4 Discussion

To the best of our knowledge, this is the first study to examine the association between LCPDLSP and DLSSDP scores—used as proxies for specific municipal long‐term care prevention efforts aimed at optimizing comprehensive care resources for older adults—and the homebound status of older adults in a large population‐based sample. After adjusting for all covariates, the multilevel logistic regression analysis indicated that older adults in municipalities with low DLSSDP scores had significantly higher odds of being homebound compared with those in municipalities with high scores. Findings from the main, supplementary stratified, and sensitivity analyses consistently suggested the potential effectiveness of specific municipal long‐term care prevention efforts, as reflected in these scores.

Previous observational studies have explored the risk factors associated with homebound status. A systematic review reported that being homebound results from the interaction between individual characteristics and structural as well as community attributes [34]. Regarding community support factors, most previous studies have concentrated on individuals who were already homebound rather than on the early prevention of homebound status [18, 19, 35]. However, preventing older adults from becoming homebound requires acknowledging the importance of social support and community participation, which foster interpersonal connections and encourage out‐of‐home activities. One previous study found that community social capital significantly influences homebound status among older adults residing in rural areas [8]. A novel aspect of our study is that it highlights the potential benefits of municipal prevention efforts—particularly those involving contributions from living support coordinators and consultative bodies—in mitigating homebound status.

This study identified a significant association between higher DLSSDP scores and a lower likelihood of being homebound, whereas LCPDLSP did not yield statistically significant results. However, our analysis did not directly test whether the odds ratios of DLSSDP and LCPDLSP scores significantly differed. In addition, as shown in Table 1; Tables S10 and S11, the incidence of homebound status was generally lower in municipalities with the highest LCPDLSP or DLSSDP scores compared with those with the lowest scores. Therefore, we cannot conclude that DLSSDP alone, rather than LCPDLSP, was effective. Nevertheless, if DLSSDP proves to be more effective than LCPDLSP, several possible mechanisms may explain this difference. Further discussions on these points are provided in Supplementary Discussion.

This study has several strengths. First, we employed the cohort design with a three‐year follow‐up, enabling the establishment of the temporal relationship between local governments' efforts and homebound status. Second, we applied rigorous analytical methods to examine the association using the LCPDLSP and DLSSDP scores, which reflect Japan's innovative long‐term care initiatives for older adults. Third, incorporating these municipal efforts uncovered patterns not apparent at the individual level and offered a more complete understanding of community‐level influences on health outcomes.

Nevertheless, this study has several limitations. First, caution is warranted when generalizing the findings—particularly to municipalities not participating in the JAGES survey and, more broadly, to non‐Japanese populations—as the analysis focused on local efforts undertaken by Japanese municipal governments. Second, because participation in the survey was voluntary, selection bias arising from non‐response or dropout may have occurred. Third, the data may be affected by response bias, as older adults with more severe homebound status might have been less likely to return the postal survey. Fourth, the possibility of reverse causality cannot be ruled out. Active engagement of older residents in community activities may also generate various needs, prompting coordinators and consultative bodies to develop resources and improve DLSSDP scores. However, the cohort study design employed in this research could substantially reduce this risk. Fifth, the final wave of the panel survey was conducted in 2022. Thus, the results might have been influenced by the COVID‐19 pandemic. If the pandemic systematically altered the effects of the 2019 LCPDLSP and DLSSDP scores, it may have biased the associations examined; however, a definitive conclusion cannot be drawn. Sixth, the data on the 2019 LCPDLSP and DLSSDP scores were available only as total values for each municipality, with no details of component indicators. Therefore, we could not assess the contribution of each component to the association between the total scores and homebound status. This limitation arises because the scores were originally compiled by the Ministry of Health, Labour and Welfare for grant allocation based on municipal prevention efforts, rather than for detailed analytical research on component‐level effects. Future studies with access to disaggregated component data are warranted to clarify the influence of each component on homebound status. Seventh, in addition to the LCPDLSP and DLSSDP, municipalities undertake various other initiatives aimed at enhancing social participation and reducing homebound status, such as utilizing social networking services and organizing targeted events. Further research is needed to clarify the association between these additional municipal efforts and homebound status. Eighth, although we adjusted for population density and municipal‐level average income, other unmeasured municipal characteristics may have influenced the association. Thus, residual confounding at the municipality level cannot be entirely excluded. However, our stratified analysis by population density suggested that the association was stronger in municipalities with lower population density than in those with high population density, where opportunities to go out are presumed to be more abundant. These findings support our hypothesis.

In conclusion, living in municipalities with higher DLSSDP scores was associated with not being homebound. Further studies are required to determine the contribution of specific component indicators within the LCPDLSP and DLSSDP scores to the prevention of homebound status.

## Author Contributions

Conceptualization: All authors. Investigation: K.Y., K.I., S.K., N.K., J.A. Methodology, Software, formal analysis, visualization, writing – original draft: DSMH, J.A. Writing – review and editing: All authors. Funding acquisition, supervision: J.A. All authors gave their final approval and agreed to be accountable for all aspects of the work.

## Conflicts of Interest

The authors declare no conflicts of interest.

## Supporting information


**Table S1:** Evaluation criteria for the 2019 long‐term care prevention and daily living support progress scores for municipalities.
**Table S2:** Evaluation criteria for the 2019 daily living support system development progress scores for municipalities.
**Table S3:** Numbers and percentages of missing variables used in multiple imputation (*n* = 89 914).
**Table S4:** Incidence of homebound status in 2022 by baseline (2019) demographic characteristics among participants who were not homebound at baseline, after multiple imputation, presented as column percentages (*n* = 89 914).
**Table S5:** Demographic characteristics of participants who were not homebound at baseline (2019), stratified by the long‐term care prevention and daily living support progress score levels after multiple imputation (*n* = 89 914).
**Table S6:** Demographic characteristics of participants who were not homebound at baseline (2019), stratified by the daily living support system development progress score levels after multiple imputation (*n* = 89 914).
**Table S7:** Incidence of homebound status in 2022 by baseline (2019) demographic characteristics among participants who were not homebound at baseline, by complete case analysis (*n* = 62 806).
**Table S8:** Association between individual‐ and municipality‐level factors and homebound status among older adults across municipalities using multilevel logistic regression by complete case analysis (*n* = 62 806).
**Table S9:** Stratified analysis by municipal population density using multilevel logistic regression after multiple imputation (*n* = 89 914).
**Table S10:** Sensitivity analysis: Association between 2020 LCPDLSP and DLSSDP scores and 2022 homebound status among older adults across municipalities, using multilevel logistic regression after multiple imputation (*n* = 89 914).
**Table S11:** Sensitivity analysis: Association between 2021 LCPDLSP and DLSSDP scores and 2022 homebound status among older adults across municipalities, using multilevel logistic regression after multiple imputation (*n* = 89 914).
**Figure S1:** Distribution of raw scores of 2019 long‐term care prevention and daily living support progress (LCPDLSP) after multiple imputation (*n* = 89 914).
**Figure S2:** Distribution of raw scores of 2019 daily living support system development progress (DLSSDP) after multiple imputation (*n* = 89 914).
**Figure S3:** Distribution of raw scores of 2020 long‐term care prevention and daily living support progress (LCPDLSP) after multiple imputation (*n* = 89 914).
**Figure S4:** Distribution of raw scores of 2020 daily living support system development progress (DLSSDP) after multiple imputation (*n* = 89 914).
**Figure S5:** Distribution of raw scores of 2021 long‐term care prevention and daily living support progress (LCPDLSP) after multiple imputation (*n* = 89 914).
**Figure S6:** Distribution of raw scores of 2021 daily living support system development progress (DLSSDP) after multiple imputation (*n* = 89 914).
**Figure S7:** Incidence of homebound status between 2019 and 2022 based on the 2019 daily living support system development progress and long‐term care prevention and daily living support progress scores, stratified by municipalities, based on complete case analysis (*n* = 62 806).

## Data Availability

The datasets used in this research are available from the corresponding author upon reasonable request. All inquiries should be directed to the data management committee via email at dataadmin.ml@jages.net.
