# Supplementary material for: Municipal Governments' Long‐Term Care Prevention Efforts and Homebound Status of Older Adults: A Three‐Year Cohort Study in Japan
Source: Geriatr Gerontol Int. 2025 Oct 15;25(11):1580–90. doi: 10.1111/ggi.70209 (PMC12584943; doi:10.1111/ggi.70209)
Supplement: Supplementary file 1 — Table S1: Evaluation criteria for the 2019 long‐term care prevention and daily living support progress scores for municipalities. Table S2: Evaluation criteria for the 2019 daily living support system development progress scores for municipalities. Table S3: Numbers and percentages of missing variables used in multiple imputation (n = 89 914). Table S4: Incidence of homebound status in 2022 by baseline (2019) demographic characteristics among participants who were not homebound at baseline, after multiple imputation, presented as column percentages (n = 89 914). Table S5: Demographic characteristics of participants who were not homebound at baseline (2019), stratified by the long‐term care prevention and daily living support progress score levels after multiple imputation (n = 89 914). Table S6: Demographic characteristics of participants who were not homebound at baseline (2019), stratified by the daily living support system development progress score levels after multiple imputation (n = 89 914). Table S7: Incidence of homebound status in 2022 by baseline (2019) demographic characteristics among participants who were not homebound at baseline, by complete case analysis (n = 62 806). Table S8: Association between individual‐ and municipality‐level factors and homebound status among older adults across municipalities using multilevel logistic regression by complete case analysis (n = 62 806). Table S9: Stratified analysis by municipal population density using multilevel logistic regression after multiple imputation (n = 89 914). Table S10: Sensitivity analysis: Association between 2020 LCPDLSP and DLSSDP scores and 2022 homebound status among older adults across municipalities, using multilevel logistic regression after multiple imputation (n = 89 914). Table S11: Sensitivity analysis: Association between 2021 LCPDLSP and DLSSDP scores and 2022 homebound status among older adults across municipalities, using multilevel logistic regression after multiple imputation ( [file GGI-25-1580-s001.docx]

**Supplementary Material**

**Municipal governments’ long-term care prevention efforts and homebound status of older adults: A three-year cohort study in Japan**

**Supplementary Methods**

**Confounders**

Individual- and municipality-level confounders were included in the analysis**.** Based on a previous study,^1^ individual characteristics comprised age, sex, living arrangements, marital status, educational attainment, equivalent annual household income, depressive symptoms, self-rated health, and number of medical diseases under care or sequelae. Age was grouped into five categories: 65–69, 70–74, 75–79, 80–84, and ≥85 years. **Sex was classified as male or female**. Living arrangements were categorized as living with other family members or living alone. Marital status was defined as having or not having a spouse. Educational attainment was dichotomized into ≥10 years or <10 years of education. Equivalent annual household income was classified into three groups: ≥4.0 million yen, ≥1.0 and <4.0 million yen, and <1.0 million yen.

Depressive symptoms were assessed using the 15-item Geriatric Depression Scale and categorized as no depression (0–4), mild depression (5–9), or severe depression (10–15). Self-rated health was assessed as good or poor. The number of medical conditions or sequelae from the 18 major diseases was grouped into four categories: 0, 1, 2, or ≥3 diseases.

At the municipality level, population density^2^ and average income^3^ were considered as confounders. Population density of habitable land, obtained from the 2019 Statistics on Prefectures, Cities, Towns, and Villages,^2^ was categorized as metropolitan (≥4,000 people/km²), urban (1,000–3,999 people/km²), or rural (<1,000 people/km²) according to a previous study.^4^ Municipality-level average income in 2019, derived from the Survey of Income Tax Rates by Tax Base Amount for fiscal year 2019,^3^ was divided into three groups based on participant tertiles: ≥3,800 thousand yen/person, 2,900–3,799 thousand yen/person, and <2,900 thousand yen/person.

**Supplementary Results**

The findings from the complete case analysis (Supplementary Tables 7 and 8; Supplementary Figure 7) were consistent with those obtained from the multiple imputation analysis (Tables 1 and 2; Figure 2).

The supplementary stratified analysis by municipal population density, conducted using multilevel logistic regression after multiple imputation, is shown in Supplementary Table 9. Overall, in municipalities with middle or low population density, the results were generally consistent with those of the main analysis: the odds ratios for being homebound tended to be higher in areas with lower DLSSDP or LCPDLSP scores. However, in high-density municipalities—where opportunities to go out are presumably more abundant—this pattern was not observed; the incidence of homebound status was lower in areas with low score levels.

The results of the sensitivity analyses using DLSSDP or LCPDLSP scores from 2020 and 2021 as explanatory variables are presented in Supplementary Tables 10 and 11, respectively. In most models, municipalities with higher DLSSDP or LCPDLSP score levels consistently exhibited a lower incidence of homebound status, although statistical significance was not uniformly observed. This trend was consistent with the findings of the main analysis.

**Supplementary Discussion**

If DLSSDP is more effective than LCPDLSP, there may be underlying mechanisms explaining this difference. One possible explanation is the variation in the content and focus of the two scoring systems. The DLSSDP places greater emphasis on the active roles of living support coordinators and consultative bodies,^5^ who are responsible for identifying local needs, building community resource networks, coordinating among stakeholders, and developing services tailored to the community context. Because they are deeply embedded within communities, the coordinators and consultative bodies can implement targeted and robust long-term care prevention strategies that align with local priorities, thereby enhancing resource development and fostering community engagement. Such efforts may encourage older adults to participate in civic activities, ultimately reducing the likelihood of becoming homebound. This interpretation is supported by a previous study conducted in China, which found that coordinated efforts by social workers and community organizations enhanced social engagement and reduced social isolation among older people. ^6^ These findings highlight the importance of community-based coordinators and organizations in addressing homebound status.

In contrast, the LCPDLSP encompasses a range of components, some of which are more closely aligned with long-term care than with the direct prevention of homebound status—such as information dissemination and the involvement of rehabilitation professionals. While these components are undoubtedly important, their impact may be indirect or delayed unless they are actively incorporated into targeted strategies for preventing homebound status.

Additionally, although not statistically significant, the odds ratio for homebound status was slightly lower in municipalities with middle LCPDLSP score levels compared with those with high levels. This result may reflect the lower incidence of homebound status in municipalities with middle LCPDLSP score levels, as shown in Table 1.

Regarding the supplementary stratified analysis by population density, the DLSSDP score was not significantly associated with homebound status in municipalities with high population density. In contrast, the LCPDLSP score showed an inverse association with homebound status in these areas. A possible explanation is that older adults living in high-density municipalities may have greater access to alternative opportunities or resources that mitigate the risk of becoming homebound, independent of LCPDLSP or DLSSDP. Such potential confounding factors may also help explain the lower odds ratio of being homebound shown in Table 2 among participants residing in municipalities with middle LCPDLSP score levels, as discussed in the previous paragraph.

Moreover, although the sensitivity analyses using LCPDLSP and DLSSDP scores from 2020 and 2021 supported the overall trend observed in the main analysis, the statistical significance of the results was not consistently stable. From a theoretical standpoint, this may be attributed to the timing of the explanatory variables. The 2019 scores, being closer to the start of the cohort study, may have shown a stronger association with the outcome compared with scores from later years. In addition, the 2020 and 2021 versions of the LCPDLSP and DLSSDP scores incorporated additional assessment indicators and revised scoring methods, which may have contributed to the observed variation in statistical significance. These findings suggest that the 2019 versions of the LCPDLSP and DLSSDP scores may have had stronger predictive validity for homebound status in 2022.

**Supplementary references:**

[1] Tsubokawa T, Shobugawa Y, Iguchi S, et al. Do community social capital and built environment associate with homebound in older adults? The JAGES Niigata study. *Journal of Epidemiology* 2022; **32**: 254-69.

[2] Statistics Bureau of Japan . Statistics on prefectures, cities, towns, and villages

[3] Ministry of Internal Affairs and Communications . Table 11: Survey of income tax rates by tax base amount for fiscal year 2019. 2020.

[4] Ide K, Jeong S, Tsuji T, et al. Suggesting indicators of Age-Friendly City: social participation and happiness, an ecological study from the JAGES. *International Journal of Environmental Research and Public Health* 2022; **19**: 5096.

[5] Japan International Cooperation Agency. Community-based Integrated Care in Japan—Suggestions for developing countries from cases in Japan—. Japan International Cooperation Agency, 2022.

[6] He Y, Wei B, Li Y. The impact of using community home-based elderly care services on older adults’ self-reported health: fresh evidence from China. *Frontiers in Public Health* 2023; **11**: 1257463.

**Supplementary Tables**

Supplementary Table Legend

Supplementary Table 1. Evaluation criteria for the 2019 Long-term Care Prevention and Daily Living Support Progress scores for municipalities

Supplementary Table 2. Evaluation criteria for the 2019 Daily Living Support System Development Progress scores for municipalities

Supplementary Table 3. Numbers and percentages of missing variables used in multiple imputation (n = 89,914)

Supplementary Table 4. Incidence of homebound status in 2022 by baseline (2019) demographic characteristics among participants who were not homebound at baseline, after multiple imputation, presented as column percentages (n = 89,914)

Supplementary Table 5. Demographic characteristics of participants who were not homebound at baseline (2019), stratified by the Long-term Care Prevention and Daily Living Support Progress score levels after multiple imputation (n = 89,914)

Supplementary Table 6. Demographic characteristics of participants who were not homebound at baseline (2019), stratified by the Daily Living Support System Development Progress score levels after multiple imputation (n = 89,914)

Supplementary Table 7. Incidence of homebound status in 2022 by baseline (2019) demographic characteristics among participants who were not homebound at baseline, by complete case analysis (n = 62,806)

Supplementary Table 8. Association between individual- and municipality-level factors and homebound status among older adults across municipalities using multilevel logistic regression by complete case analysis (n = 62,806)

Supplementary Table 9. Stratified analysis by municipal population density using multilevel logistic regression after multiple imputation (n = 89,914)

Supplementary Table 10. Sensitivity analysis: Association between 2020 LCPDLSP and DLSSDP scores and 2022 homebound status among older adults across municipalities, using multilevel logistic regression after multiple imputation (n = 89,914)

Supplementary Table 11. Sensitivity analysis: Association between 2021 LCPDLSP and DLSSDP scores and 2022 homebound status among older adults across municipalities, using multilevel logistic regression after multiple imputation (n = 89,914)

Supplementary Table 1. Evaluation criteria for the 2019 Long-term Care Prevention and Daily Living Support Progress scores for municipalities

| Indicators | Indicators of Care Prevention/ Daily Living Support Progress scores for municipalities^a^ | Allocation of scores |
| --- | --- | --- |
| **1** | Has information about the establishment and purpose of the Comprehensive Project for Long-term Care Prevention and Daily Living Support been disseminated to local residents, service providers, and other relevant community stakeholders? | Total: 6 points |
| 2 | Have estimates been made regarding the number of various services in the Long-term Care Prevention and Daily Living Support Service Project (including services with relaxed standards, community-led support, short-term intensive prevention services, and mobility support, but excluding services equivalent to the former long-term care prevention home-visit care and day-care services under preventive benefits; the same applies hereafter)?  Furthermore, have specific measures been outlined to ensure the estimated service quantity, and has the service volume for the first year of the plan been verified? | Total: 12 points |
| 3 | In the launch of various services in the Long-term Care Prevention and Daily Living Support Service Project, as well as other Daily Living Support services, have consultations been held with life support coordinators, consultative bodies, and other local stakeholders? Additionally, have opportunities been provided to verify the implementation status after the launch? | Total: 12 points |
| 4 | Based on the needs of older adults, have various services in the Long-term Care Prevention and Daily Living Support Service Project, as well as other Daily Living Support services, been established? | Total: 12 points |
| 5 | What is the number of participants aged 65 and above in community-led gathering places that contribute to long-term care prevention? (Participation rate in gathering places = actual number of participants in gathering places / elderly population, etc.)  A. Participation rate in gathering places is ○% (top 30%) B. Participation rate in gathering places is ○% (top 50%) | a. 15 points or b. 8 points |
| 6 | Is information about various community social resources, including the comprehensive project, being provided to the Community Comprehensive Support Centers, care managers, life support coordinators, and consultative bodies? | Total: 10 points |
| 7 | Has a system been established and implemented in which rehabilitation professionals are involved in long-term care prevention activities, such as through the Community Rehabilitation Support Project (a program where rehabilitation professionals provide technical advice, etc.)? | Total: 12 points |
| 8 | Are efforts being made to actively promote residents' participation in long-term care prevention activities (excluding mere dissemination and public relations)? | Total: 10 points |

^a^: Translated from Ministry of Health, Labour and Welfare. Evaluation Indicators for the Fiscal Year 2019 Grant for the Promotion of Strengthening Insurers Functions (for Municipalities). Accessed April 20th, 2025. https://www.mhlw.go.jp/content/12300000/000759279.pdf

Supplementary Table 2. Evaluation criteria for the 2019 Daily Living Support System Development Progress scores for municipalities

| Indicators | Indicators of Daily Living Support System Development Progress scores for municipalities^a^ | Allocation of scores |
| --- | --- | --- |
| **1** | Does the municipality provide support for living support coordinators?  **A. Acceptance of consultations from living support coordinators**  B. **Provision of information on local needs and information grasped by the municipality**  C. **Provision of activity information and advanced examples of coordinators in other municipalities**  D. **Explanation to local stakeholders (including support such as accompaniment)**  E. **Support for participation in local care meetings**  F. **Presentation of activity policies and contents**  G. **Inspection of activity plans of living support coordinators**  H. **Evaluation of activities of living support coordinators**  I. **Support for participation in training and information exchange meetings held by municipalities or prefectures**  J. **Others** | Each choice is worth 1 point  Multiple selections are allowed.  Total: 10 points |
| 2 | **Are living support coordinators taking specific initiatives towards the development of community resources (identifying community needs, understanding community resources, problem identification, etc.)?**  A. **Visualization of local needs and resource status, and problem identification**  B. **Requests for cooperation from various entities, such as community organizations**  C. **Networking of stakeholders**  D. **Sharing and unifying the vision and policies for the desired community**  E. **Training of life support personnel and development of services** | A to D: 2 points each  E: 4 points  Multiple selections are allowed.  Total: 12 points |
| 3 | **Are the consultative bodies taking specific initiatives towards the development of community resources (identifying community needs, understanding community resources, etc.)?**  A. **Promotion of understanding local needs, existing community resources, and visualization of information (e.g., conducting surveys and creating community resource maps)**  B. **Planning, drafting, and policy formulation (including planning related to the training of personnel for life support services, etc.)**  C. **Unification of awareness in community building** | A: 4 points  B: 5 points  C: 3 points  Multiple selections are allowed.  Total: 12 points |
| 4 | **Are specific resources being developed to meet the needs of older people through the activities of living support coordinators and consultative bodies (including strengthening existing activities and services)?** | Total: 12 points |

^a^: Translated from Ministry of Health, Labour and Welfare. Evaluation Indicators for the Fiscal Year 2019 Grant for the Promotion of Strengthening Insurers Functions (for Municipalities). Accessed April 20th, 2025. https://www.mhlw.go.jp/content/12300000/000759279.pdf

Supplementary Table 3. Numbers and percentages of missing variables used in multiple imputation (n = 89,914)

| **Variables** | **Number and percentage**  **of missing values** | |
| --- | --- | --- |
|  | **n** | **%** |
| Age | 0 | 0 |
| Sex | 0 | 0 |
| Living arrangements | 4,882 | 5.43 |
| Marital status | 1,295 | 1.44 |
| Educational attainment | 2,152 | 2.39 |
| Equivalent annual household income | 10,773 | 11.98 |
| Depressive symptoms^a^ | 12,551 | 13.96 |
| Self-rated health | 547 | 0.61 |
| Number of medical diseases under care or sequelae | 3,289 | 3.66 |
| Population density | 0 | 0 |
| Municipality-level average income | 0 | 0 |
| Daily Living Support System Development Progress scores | 0 | 0 |
| Long-term Care Prevention and Daily Living Support Progress scores | 0 | 0 |
| Homebound status in 2022 | 1,466 | 1.63 |

^a^: We used the 15-item Geriatric Depression Scale (GDS-15) to classify the depressive symptoms

Supplementary Table 4. Incidence of homebound status in 2022 by baseline (2019) demographic characteristics among participants who were not homebound at baseline, after multiple imputation, presented as column percentages (n = 89,914)

| **Characteristics** | **Incidence of homebound status in 2022** | | |
| --- | --- | --- | --- |
|  | **Going out once or more per week** | **Going out less than once per week** | **Total** |
|  | **(col%^a^)** | **(col%^a^)** | **No. (col%^a^)** |
|  | **(n = 87,407)** | **(n = 2,507)** | **(n = 89,914)** |
| **Individual-level variables** |  |  |  |
| **Age** |  |  |  |
| 65-69 | 29.0% | 16.8% | 24,150 (26.9%) |
| 70-74 | 33.1% | 22.3% | 28,419 (31.6%) |
| 75-79 | 23.8% | 25.3% | 22,269 (24.8%) |
| 80-84 | 10.9% | 21.6% | 11,196 (12.5%) |
| ≥85 | 3.2% | 14.0% | 3,880 (4.2%) |
| **Sex** |  |  |  |
| Male | 48.4% | 49.1% | 43,520 (48.4%) |
| Female | 51.6% | 50.9% | 46,394 (51.6%) |
| **Living arrangements** |  |  |  |
| Living with others | 85.9% | 84.3% | 77,229 (85.9%) |
| Living alone | 14.1% | 15.7% | 12,685 (14.1%) |
| **Marital status** |  |  |  |
| With a spouse | 76.1% | 68.2% | 68,218 (75.9%) |
| Without a spouse | 23.9% | 31.8% | 21,696 (24.1%) |
| **Educational attainment** |  |  |  |
| ≥10 years | 76.7% | 61.3% | 68,556 (76.2%) |
| <10 years | 23.3% | 38.7% | 21,358 (23.8%) |
| **Equivalent annual household income** |  |  |  |
| ≥4.0 million yen | 12.9% | 7.6% | 11,431 (12.7%) |
| ≥1.0 and <4.0 million yen | 76.6% | 70.5% | 68,757 (76.5%) |
| <1.0 million yen | 10.5% | 21.9% | 9,727 (10.8%) |
| **Depressive symptoms^b^** |  |  |  |
| No depression | 80.8% | 67.4% | 72,310 (80.4%) |
| Mild depression | 15.6% | 25.2% | 14,304 (15.9%) |
| Severe depression | 3.6% | 7.4% | 3,300 (3.7%) |
| **Self-rated health** |  |  |  |
| Good | 91.1% | 81.3% | 81,693 (90.9%) |
| Poor | 8.9% | 18.7% | 8,221 (9.1%) |
| **Number of medical diseases under care or sequelae** |  |  |  |
| 0 | 20.2% | 13.5% | 17,954 (20.0%) |
| 1 | 36.2% | 34.9% | 32,532 (36.2%) |
| 2 | 25.8% | 27.6% | 23,203 (25.8%) |
| ≥3 | 17.8% | 24.0% | 16,225 (18.0%) |
| **Municipality-level variables** |  |  |  |
| **Population density** |  |  |  |
| Metropolitan | 33.2% | 27.1% | 29,651 (33.0%) |
| Urban | 29.3% | 23.8% | 26,184 (29.1%) |
| Rural | 37.5% | 49.1% | 34,079 (37.9%) |
| **Municipality-level average income** |  |  |  |
| ≥3,800 thousand yen/person | 34.0% | 27.4% | 30,369 (33.8%) |
| ≥2,900 and <3,800 thousand yen/person | 33.0% | 27.9% | 29,506 (32.8%) |
| <2,900 thousand yen/person | 33.0% | 44.7% | 30,039 (33.4%) |
| **LCPDLSP^c^ score levels** |  |  |  |
| High | 22.1% | 21.5% | 19,811 (22.0%) |
| Middle | 43.0% | 39.0% | 38,599 (43.0%) |
| Low | 34.9% | 39.5% | 31,504 (35.0%) |
| **DLSSDP^d^ score levels** |  |  |  |
| High | 41.4% | 34.0% | 37,052 (41.2%) |
| Middle | 29.0% | 27.8% | 26,062 (29.0%) |
| Low | 29.6% | 38.2% | 26,800 (29.8%) |

^a^: Column percentage

^b^: We used the 15-item Geriatric Depression Scale (GDS-15) to classify the depressive symptoms

^c^: 2019 Long-term Care Prevention and Daily Living Support Progress scores (0-89) of the municipalities were classified into three levels: low (0-71), medium (72-75), and high (76-89), based on the tertiles of participants

^d^: 2019 Daily Living Support System Development Progress scores (0-46) of the municipalities were classified into three levels: low (0-35), medium (36-45), and high (46), based on the tertiles of participants

Supplementary Table 5. Demographic characteristics of participants who were not homebound at baseline (2019), stratified by the Long-term Care Prevention and Daily Living Support Progress score levels after multiple imputation (n = 89,914)

| **Characteristics** | **Long-term Care Prevention and Daily Living Support Progress score levels** | | | | |
| --- | --- | --- | --- | --- | --- |
|  | | **High^a^** | **Middle^a^** | **Low^a^** | **Total** |
|  |  | **(%)** | **(%)** | **(%)** | **No. (%)** |
|  |  | **(n = 19,811)** | **(n = 38,599)** | **(n = 31,504)** | **(n = 89,914)** |
| **Individual-level variables** | |  |  |  |  |
| **Homebound status in 2022** | |  |  |  |  |
| Going out once or more per week | | 97.3% | 97.5% | 96.9% | 87,407 (97.2%) |
| Going out less than once per week | | 2.7% | 2.5% | 3.1% | 2,507 (2.8%) |
| **Age** | |  |  |  |  |
| 65-69 | | 27.7% | 23.5% | 30.4% | 24,150 (26.9%) |
| 70-74 | | 31.6% | 32.2% | 30.9% | 28,419 (31.6%) |
| 75-79 | | 24.3% | 26.2% | 23.2% | 22,269 (24.8%) |
| 80-84 | | 12.1% | 13.4% | 11.5% | 11,196 (12.5%) |
| ≥85 | | 4.3% | 4.6% | 4.0% | 3,880 (4.3%) |
| **Sex** | |  |  |  |  |
| Male | | 48.1% | 49.1% | 47.8% | 43,520 (48.4%) |
| Female | | 51.9% | 50.9% | 52.2% | 46,394 (51.6%) |
| **Living arrangements** | |  |  |  |  |
| Living with others | | 86.9% | 85.4% | 85.9% | 77,229 (85.9%) |
| Living alone | | 13.2% | 14.6% | 14.1% | 12,685 (14.1%) |
| **Marital status** | |  |  |  |  |
| With a spouse | | 77.4% | 75.6% | 75.3% | 68,218 (75.9%) |
| Without a spouse | | 22.6% | 24.5% | 24.7% | 21,696 (24.1%) |
| **Educational attainment** | |  |  |  |  |
| ≥10 years | | 79.6% | 76.7% | 73.6% | 68,556 (76.3%) |
| <10 years | | 20.4% | 23.3% | 26.4% | 21,358 (23.8%) |
| **Equivalent annual household income** | |  |  |  |  |
| ≥4.0 million yen | | 12.9% | 13.8% | 11.2% | 11,431 (12.7%) |
| ≥1.0 and <4.0 million yen | | 77.0% | 76.4% | 76.2% | 68,757 (76.5%) |
| <1.0 million yen | | 10.1% | 9.8% | 12.6% | 9,727 (10.8%) |
| **Depressive symptoms^a^** | |  |  |  |  |
| No depression | | 81.5% | 80.8% | 79.3% | 72,310 (80.4%) |
| Mild depression | | 15.3% | 15.7% | 16.6% | 14,304 (15.9%) |
| Severe depression | | 3.3% | 3.6% | 4.1% | 3,300 (3.7%) |
| **Self-rated health** | |  |  |  |  |
| Good | | 91.2% | 91.1% | 90.3% | 81,693 (90.9%) |
| Poor | | 8.8% | 8.9% | 9.7% | 8,221 (9.1%) |
| **Number of medical diseases under care or sequelae** | |  |  |  |  |
| 0 | | 20.4% | 20.4% | 19.2% | 17,954 (20.0%) |
| 1 | | 36.5% | 36.0% | 36.3% | 32,532 (36.2%) |
| 2 | | 25.5% | 25.8% | 26.0% | 23,203 (25.8%) |
| ≥3 | | 17.6% | 17.9% | 18.6% | 16,225 (18.0%) |
| **Municipality-level variables** | |  |  |  |  |
| **Population density** | |  |  |  |  |
| Metropolitan | | 20.4% | 59.3% | 8.7% | 29,651 (33.0%) |
| Urban | | 51.9% | 10.6% | 37.5% | 26,184 (29.1%) |
| Rural | | 27.7% | 30.1% | 53.9% | 34,079 (37.9%) |
| **Municipality-level average income** | |  |  |  |  |
| ≥3,800 thousand yen/person | | 25.9% | 53.4% | 14.7% | 30,369 (33.8%) |
| ≥2,900 and <3,800 thousand yen/person | | 50.6% | 26.4% | 29.6% | 29,506 (32.8%) |
| <2,900 thousand yen/person | | 23.6% | 20.2% | 55.8% | 30,039 (33.4%) |

^a^: 2019 Long-term Care Prevention and Daily Living Support Progress scores (0-89) of the municipalities were classified into three levels: low (0-71), medium (72-75), and high (76-89), based on the tertiles of participants

^b^: We used the 15-item Geriatric Depression Scale (GDS-15) to classify the depressive symptoms

Supplementary Table 6. Demographic characteristics of participants who were not homebound at baseline (2019), stratified by the Daily Living Support System Development Progress score levels after multiple imputation (n = 89,914)

| **Characteristics** | **Daily Living Support System Development Progress**  **score levels** | | | | |
| --- | --- | --- | --- | --- | --- |
|  | | **High^a^** | **Middle^a^** | **Low^a^** | **Total** |
|  |  | **(%)** | **(%)** | **(%)** | **No. (%)** |
|  |  | **(n = 37,052)** | **(n = 26,062)** | **(n = 26,800)** | **(n = 89,914)** |
| **Individual-level variables** | |  |  |  |  |
| **Homebound status in 2022** | |  |  |  |  |
| Going out once or more per week | | 97.7% | 97.3% | 96.4% | 87,407 (97.2%) |
| Going out less than once per week | | 2.3% | 2.7% | 3.6% | 2,507 (2.8%) |
| **Age** | |  |  |  |  |
| 65-69 | | 21.7% | 29.7% | 31.1% | 24,150 (26.9%) |
| 70-74 | | 33.6% | 31.1% | 29.4% | 28,419 (31.6%) |
| 75-79 | | 27.0% | 24.0% | 22.4% | 22,269 (24.8%) |
| 80-84 | | 13.4% | 11.3% | 12.3% | 11,196 (12.5%) |
| ≥85 | | 4.3% | 3.9% | 4.8% | 3,880 (4.3%) |
| **Sex** | |  |  |  |  |
| Male | | 49.8% | 48.6% | 46.3% | 43,520 (48.4%) |
| Female | | 50.2% | 51.4% | 53.7% | 46,394 (51.6%) |
| **Living arrangements** | |  |  |  |  |
| Living with others | | 84.8% | 87.9% | 85.6% | 77,229 (85.9%) |
| Living alone | | 15.2% | 12.1% | 14.4% | 12,685 (14.1%) |
| **Marital status** | |  |  |  |  |
| With a spouse | | 75.6% | 77.6% | 74.6% | 68,218 (75.9%) |
| Without a spouse | | 24.4% | 22.4% | 25.4% | 21,696 (24.1%) |
| **Educational attainment** | |  |  |  |  |
| ≥10 years | | 81.4% | 75.3% | 70.0% | 68,556 (76.3%) |
| <10 years | | 18.6% | 24.7% | 30.0% | 21,358 (23.8%) |
| **Equivalent annual household income** | |  |  |  |  |
| ≥4.0 million yen | | 14.7% | 12.5% | 10.2% | 11,431 (12.7%) |
| ≥1.0 and <4.0 million yen | | 76.9% | 76.9% | 75.4% | 68,757 (76.5%) |
| <1.0 million yen | | 8.4% | 10.6% | 14.4% | 9,727 (10.8%) |
| **Depressive symptoms^a^** | |  |  |  |  |
| No depression | | 81.8% | 80.9% | 78.1% | 72,310 (80.4%) |
| Mild depression | | 15.1% | 15.5% | 17.4% | 14,304 (15.9%) |
| Severe depression | | 3.1% | 3.6% | 4.5% | 3,300 (3.7%) |
| **Self-rated health** | |  |  |  |  |
| Good | | 91.6% | 91.1% | 89.6% | 81,693 (90.9%) |
| Poor | | 8.4% | 8.9% | 10.4% | 8,221 (9.1%) |
| **Number of medical diseases under care or sequelae** | |  |  |  |  |
| 0 | | 20.6% | 19.4% | 19.7% | 17,954 (20.0%) |
| 1 | | 36.0% | 35.8% | 36.8% | 32,532 (36.2%) |
| 2 | | 25.4% | 26.3% | 25.8% | 23,203 (25.8%) |
| ≥3 | | 18.0% | 18.5% | 17.7% | 16,225 (18.0%) |
| **Municipality-level variables** | |  |  |  |  |
| **Population density** | |  |  |  |  |
| Metropolitan | | 67.3% | 7.5% | 10.2% | 29,651 (33.0%) |
| Urban | | 21.6% | 58.7% | 10.8% | 26,184 (29.1%) |
| Rural | | 11.1% | 33.8% | 79.0% | 34,079 (37.9%) |
| **Municipality-level average income** | |  |  |  |  |
| ≥3,800 thousand yen/person | | 69.2% | 7.5% | 10.2% | 30,369 (33.8%) |
| ≥2,900 and <3,800 thousand yen/person | | 19.7% | 72.6% | 12.3% | 29,506 (32.8%) |
| <2,900 thousand yen/person | | 11.1% | 19.9% | 77.5% | 30,039 (33.4%) |

^a^: 2019 Daily Living Support System Development Progress scores (0-46) of the municipalities were classified into three levels: low (0-35), medium (36-45), and high (46), based on the tertiles of participants

^b^: We used the 15-item Geriatric Depression Scale (GDS-15) to classify the depressive symptoms

Supplementary Table 7. Incidence of homebound status in 2022 by baseline (2019) demographic characteristics among participants who were not homebound at baseline, by complete case analysis (n = 62,806)

| **Characteristics** | **Incidence of homebound status in 2022** | | |
| --- | --- | --- | --- |
|  | **Going out once or more per week** | **Going out less than once per week** | **Total** |
|  | **(row%^a^)** | **(row%^a^)** | **No. (col%^b^)** |
|  | **(n = 61,292)** | **(n = 1,514)** | **(n = 62,806)** |
| **Individual-level variables** |  |  |  |
| **Age** |  |  |  |
| 65-69 | 98.6% | 1.4% | 18,036 (28.7%) |
| 70-74 | 98.4% | 1.6% | 20,595 (32.8%) |
| 75-79 | 97.4% | 2.6% | 14,951 (23.8%) |
| 80-84 | 95.3% | 4.7% | 6,988 (11.1%) |
| ≥85 | 90.5% | 9.5% | 2,236 (3.6%) |
| **Sex** |  |  |  |
| Male | 97.4% | 2.6% | 33,083 (52.7%) |
| Female | 97.8% | 2.2% | 29,723 (47.3%) |
| **Living arrangements** |  |  |  |
| Living with others | 97.6% | 2.4% | 54,535 (86.8%) |
| Living alone | 97.4% | 2.6% | 8,271 (13.2%) |
| **Marital status** |  |  |  |
| With a spouse | 97.8% | 2.2% | 49,009 (78.0%) |
| Without a spouse | 96.9% | 3.1% | 13,797 (22.0%) |
| **Educational attainment** |  |  |  |
| ≥10 years | 98.0% | 2.0% | 49,903 (79.5%) |
| <10 years | 96.1% | 4.0% | 12,903 (20.5%) |
| **Equivalent annual household income** |  |  |  |
| ≥4.0 million yen | 98.4% | 1.6% | 8,530 (13.6%) |
| ≥1.0 and <4.0 million yen | 97.8% | 2.3% | 48,569 (77.3%) |
| <1.0 million yen | 95.0% | 5.1% | 5,707 (9.1%) |
| **Depressive symptoms^c^** |  |  |  |
| No depression | 98.0% | 2.0% | 50,866 (81.0%) |
| Mild depression | 96.1% | 3.9% | 9,706 (15.5%) |
| Severe depression | 95.3% | 4.7% | 2,234 (3.6%) |
| **Self-rated health** |  |  |  |
| Good | 97.9% | 2.2% | 57,251 (91.2%) |
| Poor | 94.9% | 5.1% | 5,555 (8.8%) |
| **Number of medical diseases under care or sequelae** |  |  |  |
| 0 | 98.4% | 1.6% | 12,753 (20.3%) |
| 1 | 97.8% | 2.2% | 22,567 (35.9%) |
| 2 | 97.3% | 2.7% | 16,170 (25.8%) |
| ≥3 | 96.7% | 3.3% | 11,316 (18.0%) |
| **Municipality-level variables** |  |  |  |
| **Population density** |  |  |  |
| Metropolitan | 97.9% | 2.2% | 21,777 (34.7%) |
| Urban | 97.9% | 2.1% | 19,265 (30.7%) |
| Rural | 97.0% | 3.0% | 21,764 (34.6%) |
| **Municipality-level average income** |  |  |  |
| ≥3,800 thousand yen/person | 97.9% | 2.1% | 22,333 (35.6%) |
| ≥2,900 and <3,800 thousand yen/person | 97.8% | 2.2% | 21,570 (34.3%) |
| <2,900 thousand yen/person | 97.0% | 3.1% | 18,903 (30.1%) |
| **LCPDLSP^d^ score levels** |  |  |  |
| High | 97.6% | 2.4% | 14,081 (22.4%) |
| Middle | 97.8% | 2.3% | 27,628 (44.0%) |
| Low | 97.4% | 2.6% | 21,097 (33.6%) |
| **DLSSDP^e^ score levels** |  |  |  |
| High | 97.9% | 2.1% | 27,323 (43.5%) |
| Middle | 97.7% | 2.3% | 18,526 (29.5%) |
| Low | 97.1% | 2.9% | 16,957 (27.0%) |

^a^: Row percentage

^b^: Column percentage

^c^: We used the 15-item Geriatric Depression Scale (GDS-15) to classify the depressive symptoms

^d^: 2019 Long-term Care Prevention and Daily Living Support Progress scores (0-89) of the municipalities were classified into three levels: low (0-71), medium (72-75), and high (76-89), based on the tertiles of participants

^e^: 2019 Daily Living Support System Development Progress scores (0-46) of the municipalities were classified into three levels: low (0-35), medium (36-45), and high (46), based on the tertiles of participants

Supplementary Table 8. Association between individual- and municipality-level factors and homebound status among older adults across municipalities using multilevel logistic regression by complete case analysis (n = 62,806)

|  | **Model 1^a^** | | **Model 2^b^** | | **Model 3^c^** | | **Model 4^d^** | |
| --- | --- | --- | --- | --- | --- | --- | --- | --- |
|  | **OR^e^** | **95% CI^f^** | **OR^e^** | **95% CI^f^** | **OR^e^** | **95% CI^f^** | **OR^e^** | **95% CI^f^** |
| **Fixed Effects** |  |  |  |  |  |  |  |  |
| **Explanatory variables** |  |  |  |  |  |  |  |  |
| **LCPDLSP^g^ score levels** |  |  |  |  |  |  |  |  |
| High | 1.00 | Reference |  |  | 1.00 | Reference |  |  |
| Middle | 0.92 | 0.74; 1.14 |  |  | 0.83 | 0.68; 1.02 |  |  |
| Low | 1.11 | 0.88; 1.38 |  |  | 0.96 | 0.78; 1.18 |  |  |
| **DLSSDP^h^ score levels** |  |  |  |  |  |  |  |  |
| High | 1.00 | Reference |  |  |  |  | 1.00 | Reference |
| Middle | 1.11 | 0.88; 1.41 |  |  |  |  | 1.10 | 0.91; 1.34 |
| Low | 1.49 | 1.21; 1.84 |  |  |  |  | 1.19 | 1.02; 1.38 |
| **Individual-level confounders** |  |  |  |  |  |  |  |  |
| **Age** |  |  |  |  |  |  |  |  |
| 65-69 | 1.00 | Reference | 1.00 | Reference | 1.00 | Reference | 1.00 | Reference |
| 70-74 | 1.22 | 1.02; 1.48 | 1.16 | 0.96; 1.40 | 1.16 | 0.96; 1.40 | 1.16 | 0.96; 1.40 |
| 75-79 | 1.94 | 1.59; 2.38 | 1.70 | 1.38; 2.11 | 1.70 | 1.38; 2.11 | 1.71 | 1.38; 2.11 |
| 80-84 | 3.61 | 3.03; 4.30 | 3.02 | 2.47; 3.69 | 3.02 | 2.47; 3.69 | 3.02 | 2.47; 3.69 |
| ≥85 | 7.56 | 6.13; 9.33 | 5.94 | 4.83; 7.31 | 5.94 | 4.83; 7.32 | 5.96 | 4.84; 7.33 |
| **Sex** |  |  |  |  |  |  |  |  |
| Male | 1.00 | Reference | 1.00 | Reference | 1.00 | Reference | 1.00 | Reference |
| Female | 0.87 | 0.75; 1.00 | 0.84 | 0.74; 0.96 | 0.84 | 0.74; 0.96 | 0.84 | 0.74; 0.97 |
| **Living arrangements** |  |  |  |  |  |  |  |  |
| Living with others | 1.00 | Reference | 1.00 | Reference | 1.00 | Reference | 1.00 | Reference |
| Living alone | 1.11 | 0.91; 1.35 | 0.78 | 0.62; 0.98 | 0.78 | 0.62; 0.98 | 0.78 | 0.62; 0.98 |
| **Marital status** |  |  |  |  |  |  |  |  |
| With a spouse | 1.00 | Reference | 1.00 | Reference | 1.00 | Reference | 1.00 | Reference |
| Without a spouse | 1.40 | 1.21; 1.63 | 1.24 | 1.06; 1.44 | 1.23 | 1.06; 1.44 | 1.23 | 1.06; 1.44 |
| **Educational attainment** |  |  |  |  |  |  |  |  |
| ≥10 years | 1.00 | Reference | 1.00 | Reference | 1.00 | Reference | 1.00 | Reference |
| <10 years | 2.02 | 1.72; 2.36 | 1.33 | 1.14; 1.56 | 1.34 | 1.14; 1.56 | 1.33 | 1.14; 1.56 |
| **Equivalent annual household income** |  |  |  |  |  |  |  |  |
| ≥4.0 million yen | 1.00 | Reference | 1.00 | Reference | 1.00 | Reference | 1.00 | Reference |
| ≥1.0 and <4.0 million yen | 1.43 | 1.22; 1.68 | 1.26 | 1.08; 1.48 | 1.26 | 1.08; 1.48 | 1.26 | 1.08; 1.45 |
| <1.0 million yen | 3.17 | 2.63; 3.81 | 2.08 | 1.73; 2.51 | 2.09 | 1.74; 2.51 | 2.08 | 1.73; 2.50 |
| **Depressive symptoms^i^** |  |  |  |  |  |  |  |  |
| Non | 1.00 | Reference | 1.00 | Reference | 1.00 | Reference | 1.00 | Reference |
| Mild | 1.94 | 1.69; 2.22 | 1.64 | 1.40; 1.92 | 1.64 | 1.40; 1.92 | 1.64 | 1.40; 1.92 |
| Severe | 2.35 | 1.93; 2.87 | 1.73 | 1.36; 2.19 | 1.73 | 1.36; 2.19 | 1.72 | 1.36; 2.19 |
| **Self-rated health** |  |  |  |  |  |  |  |  |
| Fair | 1.00 | Reference | 1.00 | Reference | 1.00 | Reference | 1.00 | Reference |
| Poor | 2.40 | 2.07; 2.78 | 1.68 | 1.45; 1.96 | 1.68 | 1.45; 1.96 | 1.69 | 1.45; 1.96 |
| **Number of medical diseases under care or sequelae** |  |  |  |  |  |  |  |  |
| 0 | 1.00 | Reference | 1.00 | Reference | 1.00 | Reference | 1.00 | Reference |
| 1 | 1.37 | 1.11; 1.69 | 1.14 | 0.92; 1.41 | 1.14 | 0.92; 1.41 | 1.14 | 0.93; 1.41 |
| 2 | 1.66 | 1.35; 2.03 | 1.25 | 1.01; 1.54 | 1.25 | 1.01; 1.54 | 1.25 | 1.01; 1.54 |
| ≥3 | 2.02 | 1.56; 2.62 | 1.28 | 0.98; 1.68 | 1.28 | 0.98; 1.68 | 1.28 | 0.98; 1.68 |
| **Municipality-level confounders** |  |  |  |  |  |  |  |  |
| **Population density** |  |  |  |  |  |  |  |  |
| Metropolitan | 1.00 | Reference | 1.00 | Reference | 1.00 | Reference | 1.00 | Reference |
| Urban | 0.99 | 0.80; 1.23 | 0.90 | 0.61; 1.34 | 0.81 | 0.55; 1.19 | 0.89 | 0.61; 1.30 |
| Rural | 1.48 | 1.26; 1.74 | 1.10 | 0.71; 1.70 | 1.02 | 0.67; 1.56 | 1.05 | 0.68; 1.62 |
| **Municipality-level average income** |  |  |  |  |  |  |  |  |
| ≥3,800 thousand yen/person | 1.00 | Reference | 1.00 | Reference | 1.00 | Reference | 1.00 | Reference |
| ≥2,900 and <3,800 thousand yen/person | 1.07 | 0.86; 1.32 | 1.06 | 0.70; 1.60 | 1.11 | 0.75; 1.64 | 1.02 | 0.71; 1.48 |
| <2,900 thousand yen/person | 1.57 | 1.28; 1.92 | 1.23 | 0.75; 1.99 | 1.25 | 0.78; 2.01 | 1.16 | 0.73; 1.83 |
|  |  |  |  |  |  |  |  |  |
| **VPC^j^ (%)** |  | | 1.04 | | 0.86 | | 0.96 | |
| ^a^: Univariable Model | | | | | | | | |
| ^b^: Adjusted for age, sex, living arrangements, marital status, educational attainment, equivalent annual household income, depressive symptoms, self-rated health, number of medical diseases under care or sequelae, population density, municipality-level average income. | | | | | | | | |
| ^c^: Adjusted for age, sex, living arrangements, marital status, educational attainment, equivalent annual household income, depressive symptoms, self-rated health, number of medical diseases under care or sequelae, population density, and municipality-level average income. 2019 Long-term Care Prevention and Daily Living Support Progress score levels were included as explanatory variables. | | | | | | | | |
| ^d^: Adjusted for age, sex, living arrangements, marital status, educational attainment, equivalent annual household income, depressive symptoms, self-rated health, number of medical diseases under care or sequelae, population density, and municipality-level average income. 2019 Daily Living Support System Development Progress scores levels were included as explanatory variables. | | | | | | | | |
| ^e^: Odds Ratio | | | | | | | | |
| ^f^: Confidence Interval | | | | | | | | |
| ^g^: Long-term Care Prevention and Daily Living Support Progress | | | | | | | | |
| ^h^: Daily Living Support System Development Progress | | | | | | | | |
| ^i^: We used the 15-item Geriatric Depression Scale (GDS-15) to classify the depressive symptoms | | | | | | | | |
| ^j^: Variance Partition Coefficient | | | | | | | | |

Supplementary Table 9. Stratified analysis by municipal population density using multilevel logistic regression after multiple imputation (n = 89,914)

|  | **High population density^a^ (n = 29,651)** | | | | | | |
| --- | --- | --- | --- | --- | --- | --- | --- |
|  | **Incidence of homebound status^d^** | **Model 1^e^** | | **Model 2^f^** | | **Model 3^g^** | |
|  |  |  |  |  |  |  |  |
|  | **%** | **OR^h^** | **95% CI^i^** | **OR^h^** | **95% CI^i^** | **OR^h^** | **95% CI^i^** |
| **LCPDLSP^j^ score levels** |  |  |  |  |  |  |  |
| High | 2.46 | 1.00 | Reference | 1.00 | Reference |  |  |
| Middle | 2.29 | 0.93 | 0.82; 1.05 | 0.84 | 0.74; 0.95 |  |  |
| Low | 1.99 | 0.81 | 0.73; 0.89 | 0.83 | 0.72; 0.94 |  |  |
| **DLSSDP^k^ score levels** |  |  |  |  |  |  |  |
| High | 2.32 | 1.00 | Reference |  |  | 1.00 | Reference |
| Middle | 2.32 | 1.00 | 0.92; 1.09 |  |  | 1.04 | 0.96; 1.12 |
| Low | 1.99 | 0.86 | 0.78; 0.94 |  |  | 0.96 | 0.88; 1.05 |
|  | **Middle population density^b^ (n = 26,184)** | | | | | | |
| **LCPDLSP^j^ score levels** |  |  |  |  |  |  |  |
| High | 2.36 | 1.00 | Reference | 1.00 | Reference |  |  |
| Middle | 2.12 | 0.91 | 0.73; 1.14 | 0.87 | 0.61; 1.22 |  |  |
| Low | 2.27 | 0.95 | 0.63; 1.42 | 0.82 | 0.55; 1.23 |  |  |
| **DLSSDP^k^ score levels** |  |  |  |  |  |  |  |
| High | 1.78 | 1.00 | Reference |  |  | 1.00 | Reference |
| Middle | 2.47 | 1.33 | 0.96; 1.85 |  |  | 1.31 | 1.01; 1.69 |
| Low | 2.65 | 1.42 | 1.02; 1.97 |  |  | 1.67 | 1.32; 2.12 |
|  | **Low population density^c^ (n = 34,079)** | | | | | | |
| **LCPDLSP^j^ score levels** |  |  |  |  |  |  |  |
| High | 3.58 | 1.00 | Reference | 1.00 | Reference |  |  |
| Middle | 3.14 | 0.9 | 0.73; 1.11 | 0.90 | 0.71; 1.14 |  |  |
| Low | 3.94 | 1.12 | 0.95; 1.31 | 1.13 | 0.93; 1.34 |  |  |
| **DLSSDP^k^ score levels** |  |  |  |  |  |  |  |
| High | 3.23 | 1.00 | Reference |  |  | 1.00 | Reference |
| Middle | 3.09 | 0.98 | 0.77; 1.24 |  |  | 1.05 | 0.80; 1.39 |
| Low | 3.91 | 1.28 | 1.07; 1.53 |  |  | 1.20 | 0.98; 1.47 |

^a^: high population density: comprising 7 municipalities

^b^: middle population density: comprising 13 municipalities

^c^: low population density: comprising 29 municipalities

^d^: Incidence of homebound status between 2019 and 2022

^e^: Univariable Model

^f^: Adjusted for age, sex, living arrangements, marital status, educational attainment, equivalent annual household income, depressive symptoms, self-rated health, number of medical diseases under care or sequelae, population density, municipality-level average income. 2019 Long-term Care Prevention and Daily Living Support Progress score levels were included as explanatory variables.

^g^: Adjusted for age, sex, living arrangements, marital status, educational attainment, equivalent annual household income, depressive symptoms, self-rated health, number of medical diseases under care or sequelae, population density, municipality-level average income. 2019 Daily Living Support System Development Progress score levels were included as explanatory variables.

^h^: Odds Ratio

^i^: Confidence Interval

^j^: 2019 Preventive Care and Daily Living Support Progress

^k^: 2019 Daily Living Support System Development Progress

Supplementary Table 10. Sensitivity analysis: Association between 2020 LCPDLSP and DLSSDP scores and 2022 homebound status among older adults across municipalities, using multilevel logistic regression after multiple imputation (n = 89,914)

|  | **Distribution^a^** | **Incidence of homebound status^b^** | **Model 1^c^** | | **Model 2^d^** | | **Model 3^e^** | |
| --- | --- | --- | --- | --- | --- | --- | --- | --- |
|  |  |  |  |  |  |  |  |  |
|  | **No. (col%^f^)**  **(n = 89,914)** | **%** | **OR^g^** | **95% CI^h^** | **OR^g^** | **95% CI^h^** | **OR^g^** | **95% CI^h^** |
|  |  |  |  |  |  |  |  |  |
| **LCPDLSP score^i^ levels** |  |  |  |  |  |  |  |  |
| High | 31,939 (35.5%) | 2.47 | 1.00 | Reference | 1.00 | Reference |  |  |
| Middle | 28,857 (32.1%) | 2.82 | 1.26 | 0.98; 1.63 | 1.20 | 1.004; 1.43 |  |  |
| Low | 29,118 (32.4%) | 3.10 | 1.36 | 1.07; 1.74 | 1.15 | 0.98; 1.36 |  |  |
|  |  |  |  |  |  |  |  |  |
| **DLSSDP score^j^ levels** |  |  |  |  |  |  |  |  |
| High | 28,270 (31.4%) | 2.49 | 1.00 | Reference |  |  | 1.00 | Reference |
| Middle | 29,139 (32.4%) | 2.83 | 1.25 | 0.91; 1.72 |  |  | 1.01 | 0.83; 1.23 |
| Low | 32,505 (36.2%) | 3.01 | 1.23 | 0.94; 1.62 |  |  | 1.05 | 0.89; 1.25 |

^a^: Distribution of 2020 LCPDLSP and DLSSDP score levels across the study sample (n = 89,914)

^b^: Incidence of homebound status between 2019 and 2022

^c^: Univariable Model

^d^: Adjusted for age, sex, living arrangements, marital status, educational attainment, equivalent annual household income, depressive symptoms, self-rated health, number of medical diseases under care or sequelae, population density, municipality-level average income. 2020 Long-term Care Prevention and Daily Living Support Progress score levels were included as explanatory variables.

^e^: Adjusted for age, sex, living arrangements, marital status, educational attainment, equivalent annual household income, depressive symptoms, self-rated health, number of medical diseases under care or sequelae, population density, municipality-level average income. 2020 Daily Living Support System Development Progress score levels were included as explanatory variables.

^f^: Column percentage

^g^: Odds Ratio

^h^: Confidence Interval

^i^: 2020 Long-term Care Prevention and Daily Living Support Progress scores (0-450) of the municipalities were classified into three levels: low (0-212), medium (213-274), and high (275-450), based on the tertiles of participants

^j^: 2020 Daily Living Support System Development Progress scores (0-85) of the municipalities were classified into three levels: low (0-53), medium (54-63), and high (64-85), based on the tertiles of participants

Supplementary Table 11. Sensitivity analysis: Association between 2021 LCPDLSP and DLSSDP scores and 2022 homebound status among older adults across municipalities, using multilevel logistic regression after multiple imputation (n = 89,914)

|  | **Distribution^a^** | **Incidence of homebound status^b^** | **Model 1^c^** | | **Model 2^d^** | | **Model 3^e^** | |
| --- | --- | --- | --- | --- | --- | --- | --- | --- |
|  |  |  |  |  |  |  |  |  |
|  | **No. (col%^f^)**  **(n = 89,914)** | **%** | **OR^g^** | **95% CI^h^** | **OR^g^** | **95% CI^h^** | **OR^g^** | **95% CI^h^** |
|  |  |  |  |  |  |  |  |  |
| **LCPDLSP score^i^ levels** |  |  |  |  |  |  |  |  |
| High | 32,693 (36.4%) | 2.59 | 1.00 | Reference | 1.00 | Reference |  |  |
| Middle | 28,694 (31.9%) | 2.77 | 1.08 | 0.83; 1.42 | 1.10 | 0.93; 1.30 |  |  |
| Low | 28,527 (31.7%) | 3.03 | 1.21 | 0.94; 1.55 | 1.08 | 0.91; 1.28 |  |  |
|  |  |  |  |  |  |  |  |  |
| **DLSSDP score^j^ levels** |  |  |  |  |  |  |  |  |
| High | 34,385 (38.2%) | 2.47 | 1.00 | Reference |  |  | 1.00 | Reference |
| Middle | 31,528 (35.1%) | 2.94 | 1.25 | 0.95; 1.65 |  |  | 0.94 | 0.77; 1.16 |
| Low | 24,001 (26.7%) | 3.04 | 1.28 | 0.96; 1.71 |  |  | 1.04 | 0.87; 1.25 |

^a^: Distribution of 2021 LCPDLSP and DLSSDP score levels across the study sample (n = 89,914)

^b^: Incidence of homebound status between 2019 and 2022

^c^: Univariable Model

^d^: Adjusted for age, sex, living arrangements, marital status, educational attainment, equivalent annual household income, depressive symptoms, self-rated health, number of medical diseases under care or sequelae, population density, municipality-level average income. 2021 Long-term Care Prevention and Daily Living Support Progress score levels were included as explanatory variables.

^e^: Adjusted for age, sex, living arrangements, marital status, educational attainment, equivalent annual household income, depressive symptoms, self-rated health, number of medical diseases under care or sequelae, population density, municipality-level average income. 2021 Daily Living Support System Development Progress score levels were included as explanatory variables.

^f^: Column percentage

^g^: Odds Ratio

^h^: Confidence Interval

^i^: 2021 Long-term Care Prevention and Daily Living Support Progress scores (0-450) of the municipalities were classified into three levels: low (0-219), medium (220-324), and high (325-450), based on the tertiles of participants

^j^: 2021 Daily Living Support System Development Progress scores (0-85) of the municipalities were classified into three levels: low (0-50), medium (51-62), and high (63-85), based on the tertiles of participants

**Supplementary Figures**

Supplementary Figure Legend

Supplementary Figure 1. Distribution of raw scores of 2019 Long-term Care Prevention and Daily Living Support Progress (LCPDLSP) after multiple imputation (n = 89,914)Supplementary Figure 2. Distribution of raw scores of 2019 Daily Living Support System Development Progress (DLSSDP) after multiple imputation (n = 89,914)

Supplementary Figure 3. Distribution of raw scores of 2020 Long-term Care Prevention and Daily Living Support Progress (LCPDLSP) after multiple imputation (n = 89,914)
Supplementary Figure 4. Distribution of raw scores of 2020 Daily Living Support System Development Progress (DLSSDP) after multiple imputation (n = 89,914)

Supplementary Figure 5. Distribution of raw scores of 2021 Long-term Care Prevention and Daily Living Support Progress (LCPDLSP) after multiple imputation (n = 89,914)
Supplementary Figure 6. Distribution of raw scores of 2021 Daily Living Support System Development Progress (DLSSDP) after multiple imputation (n = 89,914)

Supplementary Figure 7. Incidence of homebound status between 2019 and 2022 based on the 2019 Daily Living Support System Development Progress and Long-term Care Prevention and Daily Living Support Progress scores, stratified by municipalities, based on complete case analysis (n = 62,806)

Supplementary Figure 1. Raw scores of 2019 Long-term Care Prevention and Daily Living Support Progress (LCPDLSP) after multiple imputation (n = 89,914)

Supplementary Figure 2. Raw scores of 2019 Daily Living Support System Development Progress (DLSSDP) after multiple imputation (n = 89,914)

Supplementary Figure 3. Raw scores of 2020 Long-term Care Prevention and Daily Living Support Progress (LCPDLSP) after multiple imputation (n = 89,914)

Supplementary Figure 4. Raw scores of 2020 Daily Living Support System Development Progress (DLSSDP) after multiple imputation (n = 89,914)

Supplementary Figure 5. Raw scores of 2021 Long-term Care Prevention and Daily Living Support Progress (LCPDLSP) after multiple imputation (n = 89,914)

Supplementary Figure 6. Raw scores of 2021 Daily Living Support System Development Progress (DLSSDP) after multiple imputation (n = 89,914)

Supplementary Figure 7. Incidence of homebound status between 2019 and 2022 based on the 2019 Daily Living Support System Development Progress and Long-term Care Prevention and Daily Living Support Progress scores, stratified by municipalities, based on complete case analysis (n = 62,806)
